# Arabidopsis LFR, a SWI/SNF complex component, interacts with ICE1 and activates *ICE1* and *CBF3* expression in cold acclimation

**DOI:** 10.3389/fpls.2023.1097158

**Published:** 2023-03-21

**Authors:** Tian Ma, Shuge Wang, Cunyi Sun, Jiawang Tian, Hong Guo, Sujuan Cui, Hongtao Zhao

**Affiliations:** Hebei Key Laboratory of Molecular and Cellular Biology, Key Laboratory of Molecular and Cellular Biology of Ministry of Education, Hebei Research Center of the Basic Discipline of Cell Biology, Hebei Collaboration Innovation Center for Cell Signaling, College of Life Science, Hebei Normal University, Hebei, China

**Keywords:** Arabidopsis, cold acclimation, chromatin remodeling, SWI/SNF, LFR, ICE1, *CBFs*

## Abstract

Low temperatures restrict the growth and geographic distribution of plants, as well as crop yields. Appropriate transcriptional regulation is critical for cold acclimation in plants. In this study, we found that the mutation of Leaf and flower related (LFR), a component of SWI/SNF chromatin remodeling complex (CRC) important for transcriptional regulation in Arabidopsis (*Arabidopsis thaliana*), resulted in hypersensitivity to freezing stress in plants with or without cold acclimation, and this defect was successfully complemented by *LFR*. The expression levels of *CBFs* and *COR* genes in cold-treated *lfr-1* mutant plants were lower than those in wild-type plants. Furthermore, LFR was found to interact directly with ICE1 in yeast and plants. Consistent with this, LFR was able to directly bind to the promoter region of *CBF3*, a direct target of ICE1. LFR was also able to bind to *ICE1* chromatin and was required for *ICE1* transcription. Together, these results demonstrate that LFR interacts directly with ICE1 and activates *ICE1* and *CBF3* gene expression in response to cold stress. Our work enhances our understanding of the epigenetic regulation of cold responses in plants.

## Introduction

Cold stress profoundly limits the growth, development, and geographical distribution of plants, and it can significantly threaten crop production and quality (Shi et al., 2018; Ding et al., 2020; Ding and Yang, 2022). However, plants have evolved precise cellular and molecular mechanisms to improve their chance of survival under extreme low temperature conditions (Shi et al., 2018; Ding and Yang, 2022). Understanding how plants adapt to and tolerate cold stress by sensing, transducing, and responding to cold signals is paramount for the breeding of climate-resilient crops and has long been a topic of interest (Shi et al., 2018; Chen et al., 2021). The transmembrane protein COLD1 was identified as a sensor that perceives cold signals and forms a complex with G-protein α subunit1 (RGA1) to mediate cold-induced influx of intracellular Ca^2+^, subsequently leading to the expression of cold-regulated (*COR*) genes in rice (*Oryza sativa*) (Ma et al., 2015; Shi et al., 2018). *COR* genes, including *COR15A* and *Responsive to Desiccation 29*, encode proteins that can stabilize membranes against freezing-induced injury (Jaglo-Ottosen et al., 1998; Thomashow, 1999). C-repeat binding factors (CBFs), also known as dehydration-responsive element (DRE)-binding factors (DREBs), are key transcription factors (TFs) that bind to CRT/DRE motifs in the promoters of target *COR* genes, thus inducing their expression (Stockinger et al., 1997; Gilmour et al., 1998; Jaglo-Ottosen et al., 1998; Liu et al., 1998; Song et al., 2021). *CBFs* are themselves rapidly upregulated by non-freezing low temperatures (Gilmour et al., 1998). Over the past 20 years, multiple TFs have been shown to regulate *CBF* expression (Shi et al., 2018). For example, inducer of CBF expression 1 (ICE1) is the one of the well-characterized transcriptional activators of *CBF* genes (Chinnusamy et al., 2003; Kim et al., 2015; Shi et al., 2018; Ding et al., 2019).

ICE1 is a MYC-like basic helix-loop-helix (bHLH) TF that binds directly to a MYC recognition sequence (CANNTG) in the promoters of *CBF* genes under conditions of cold stress (Chinnusamy et al., 2003; Kim et al., 2015; Tang et al., 2020). In contrast to *CBFs* and *CORs*, *ICE1* is a constitutively expressed gene with no obvious response to cold stress at the transcriptional level (Chinnusamy et al., 2003; Shi et al., 2018). However, multiple post-translational modifications, including ubiquitination (Dong et al., 2006), sumoylation (Miura et al., 2007), and phosphorylation (Ding et al., 2015; Li et al., 2017; Zhao et al., 2017; Ye et al., 2019), play fundamental roles in the lifespan and turnover of ICE1 at low temperatures (Shi et al., 2018). Interacting partners that are important for the regulation of ICE1 transcriptional activity toward *CBFs* have also been reported. For example, MYB15 (Agarwal et al., 2006), JASMONATE ZIM-DOMAIN proteins (Hu et al., 2013), and MYC67 and MYC70 (Ohta et al., 2018) were separately isolated as ICE1 interactors; however, they mainly inhibit *CBF* gene transcription. Interacting factors that are important for ICE1 to activate downstream *CBFs* are largely unknown.

ATP-dependent chromatin remodeling complexes (CRCs) activate or inhibit transcription by modifying the architecture of chromatin (Clapier and Cairns, 2009; Ho and Crabtree, 2010; Hargreaves and Crabtree, 2011; Clapier et al., 2017; Yang et al., 2022). For example, the SWItch/Sucrose Non-Fermentable (SWI/SNF) CRC uses energy from ATP hydrolysis to change the structure and position of nucleosomes at target genes to activate or repress transcription; this represents a conserved transcriptional regulatory mechanism across species (Hirschhorn et al., 1992; Peterson and Herskowitz, 1992; Khavari et al., 1993; Chiba et al., 1994; Sarnowska et al., 2016; Ojolo et al., 2018; Shu et al., 2021). In the model plant Arabidopsis (*Arabidopsis thaliana*), the SWI/SNF CRC is a multi-subunit machine containing four core catalytic ATPases [BRAHMA (BRM), SPLAYED (SYD), Chromatin Remodeling Factor 12/MINUSCULE 1 (CHR12/MINU1), and CHR23/MINU2] (Wagner and Meyerowitz, 2002; Farrona et al., 2004; Bezhani et al., 2007; Sang et al., 2012), a single SNF5 subunit called BUSHY (BSH) (Brzeski et al., 1999), four SWI3 proteins (SWI3A, SWI3B, SWI3C, and SWI3D) (Sarnowski et al., 2005), two SWI/SNF ASSOCIATED PROTEINS 73 (SWP73A and SWP73B) (Jégu et al., 2014; Sacharowski et al., 2015; Jégu et al., 2017), two ACTIN-RELATED PROTEINS (ARP4 and ARP7) (Kandasamy et al., 2003), and a single Leaf and Flower Related (LFR) protein (Wang et al., 2009; Lin et al., 2021). In addition, two homologous BRM‐interacting proteins (BRIP1 and BRIP2) (Yu et al., 2020), three bromodomain‐containing proteins (BRD1, BRD2, and BRD13) (Jarończyk et al., 2021; Yu et al., 2021), ANGUSTIFOLIA3 (Vercruyssen et al., 2014), and two TRIPLE PHD FINGERS proteins (TPF1 and TFP2) (Diego-Martin et al., 2022) have been identified as components of the SWI/SNF CRC.

Components of the SWI/SNF CRC are involved in various biological processes, including plant development, phase transitions, DNA methylation, transposon silencing, pre-miRNA processing, and DNA double-strand breaks (Sarnowska et al., 2016; Ojolo et al., 2018; Thouly et al., 2020). The molecular mechanisms of BRM or SWI3B were unraveled in abscisic acid pathway (Saez et al., 2008; Han et al., 2012; Peirats-Llobet et al., 2016) and environmental stress response, such as salt stress (Vicente et al., 2017) and heat-shock (HS) stress (Brzezinka et al., 2016). Recently, SWI3C was reported to bind and alter the nucleosome occupancy and expression of key factors in cold acclimation, including *ICE1*, *MYB15*, and *CBF1*, under low temperature conditions, revealing a role for SWI/SNF components in cold acclimation (Gratkowska-Zmuda et al., 2020). However, which TFs play important roles in recruiting SWI/SNF complexes to target genes during cold acclimation is unknown.

The *LFR* gene is essential for leaf and flower development in Arabidopsis (Wang et al., 2012; Lin et al., 2018; Lin et al., 2021). *LFR* encodes an Armadillo (ARM)-repeat domain-containing protein that is predicted to be a subunit of the SWI/SNF CRC (Wang et al., 2009; Lin et al., 2021; Diego-Martin et al., 2022; Shang and He, 2022). In this study, we found that the disruption of *LFR* led to reduced freezing tolerance in Arabidopsis. We demonstrated that LFR interacts directly with ICE1 in yeast and plant. Meanwhile, chromatin immunoprecipitation (ChIP)-qPCR and Real Time Quantitative PCR (RT-qPCR) assays demonstrated that LFR can directly bind to and activate the transcription of *CBF3*, an ICE1 target gene, and, in turn, the *COR15A* gene. Moreover, *ICE1* is a direct target of LFR and LFR is required for *ICE1* expression. Together, our results show that LFR directly interacts with ICE1 and activates *CBFs* expression, revealing the molecular mechanism of the SWI/SNF CRC during cold acclimation in plants.

## Materials and methods

### Plant materials and growth conditions

The wild-type (WT) and mutant Arabidopsis plants used in this study were of the Columbia-0 (Col-0) background. The *lfr-1* and *lfr-2/+* mutants are described elsewhere (Wang et al., 2009). *ice1-2* (SALK_003155; Kanaoka et al., 2008) was a gift from Prof. Shuhua Yang’s laboratory. Seeds were surface-sterilized in 75% ethanol and 1% NaClO, washed three times with sterile water, plated on half-strength Murashige and Skoog (1/2 MS) medium with 1% sucrose and 0.36% agar, and stratified at 4°C in the dark for 3 days. All plants were grown under long-day conditions (16 h of light/8 h of dark) at 22°C.

### Plasmid construction and plant transformation

To map the domains of ICE1 (*AT3G26744*) that are involved in its interaction with LFR, full-length and truncated versions of ICE1 were fused to the GAL4 binding domain (BD) in pGBKT7. Meanwhile, LFR was fused to the GAL4 activation domain (AD) in pGADT7 (Lin et al., 2018). For our Bimolecular Fluorescence Complementation (BiFC) and firefly luciferase (LUC) complementation imaging (LCI) experiments, the full-length coding sequence (CDS) of ICE1 and LFR was amplified by PCR from Col-0 cDNA, cloned into pENTRY/D/SD-TOPO (Takara Bio Inc., Shiga, Japan), and then fused with the C-terminal half of cyan fluorescent protein (CFP) (cCFP), the C-terminal half of LUC (cLUC), the N-terminal half of yellow fluorescent protein (YFP) (nYFP), or the N-terminal half of LUC (nLUC). The full-length CDS of LFR was fused to nYFP (Lin et al., 2018).

To obtain the construct of *pLFR : LFR-enhancedYFP* (*eYFP*) and *pLFR : LFR*-3*FLAG*, the native promoter of *LFR* (1600 bp), the genomic sequence of *LFR*, and the coding sequences of *eYFP* and 3×*FLAG* were amplified and digested using appropriate restriction endonucleases and subsequently cloned into p*CAMBIA1300*. After being verified by DNA sequencing, the plasmid was introduced into *lfr-1* and/or *lfr-2*/+ by *Agrobacterium tumefaciens*-mediated floral dip transformation (Clough and Bent, 1998). The homologous transgenic plants of *pLFR : LFR-eYFP* in *lfr-2* background (*pLFR : LFR*-*eYFP*/*lfr-*2) and *pLFR : LFR*-3*FLAG* in *lfr-1* and *lfr-2* background (*pLFR : LFR*-3*FLAG*/*lfr-1* and *pLFR : LFR*-3*FLAG*/*lfr-2*) were identified in the T4 generation and used for further phenotypic observation.

### RNA isolation and RT-qPCR analysis

Total RNA was extracted from WT and mutant plants using Trizol reagent (Takara). First-strand cDNA was prepared from 500 ng of total RNA using a PrimeScipt RT Reagent Kit (Takara) and quantified using a CFX96™ Connect Real-Time PCR System (Bio-Rad, Hercules, CA, USA) with a SYBR Green Kit (Takara). The *eIF4A1* gene was used as an internal control. The primers used are listed in **Supplementary Table S1**.

### Freezing tolerance assay

A freezing tolerance assay was performed as described previously (Kim et al., 2015). Approximately 36 plants grown on 1/2 MS medium for 10 days were subjected to freezing conditions; the temperature was set to decrease 1°C/h until it reached -6°C (seedlings did not undergo cold acclimation before freezing) or -9°C (seedlings underwent cold acclimation at 4°C for 4 days before freezing). The plants were then incubated at 4°C overnight in the dark. Next, the plants were allowed to recover for 2 days at 22°C.

### Chlorophyll content analysis

The chlorophyll content measurement was performed as described previously (Arnon, 1949; Mostofa et al., 2015). After freezing treatment, the seedlings were allowed to recover for three days at 22°C. Approximately 0.5 g of seedlings from a pool of plants from different plates were dipped in 50 mL aqueous acetone (80%) at 4°C in the dark for 48 h. The light absorption by acetone extracts of chlorophyll at 663 nm and 645 nm are detected in a spectrophotometer. The chlorophyll content was calculated as (20.2 × D645 + 8.02 × D663) × V/(1000 × W); where V = volume of 80% (v/v) acetone (mL), W = weight of sample (g).

### Yeast two-hybrid assays

Y2H assays were carried out using the yeast GAL4 system following a standard protocol (Clontech Laboratories, Inc., Mountain View, CA, USA). To verify its interaction with LFR, truncated versions of ICE1 [ICE1-2 (the C-terminal region of ICE1, corresponding to amino acids 298–494) and ICE1-3 (the C-terminal region of ICE1, corresponding to amino acids 358–494)] were cloned into pGBKT7 and LFR was fused to the AD in pGADT7. Following verification of the constructs by DNA sequencing, pairs of the plasmids were co-transformed into yeast strain AH109, followed by incubation at 30°C for 3 days. The co-transformed yeast cells were grown on solid medium lacking leucine (Leu) and tryptophan (Trp) (SD/-L-W). Only when the two proteins interacted with each other could the co-transformed yeast cells grow on solid medium lacking Leu, Trp, histidine (His), and adenine (Ade) (SD/-L-W-H-A).

### BiFC and firefly LCI assays

BiFC and LCI assays were performed using transiently transformed tobacco leaves (Chen et al., 2008; Ou et al., 2011). For the BiFC assay, cCFP-ICE1 and nYFP-LFR constructs were co-transformed and co-expressed for about 48 h in tobacco epidermal cells. Fluorescent green fluorescent protein (GFP) (excitation 488 nm; emission 500–550 nm) and 4’,6-diamidino-2-phenylindole (DAPI) (excitation 405 nm; emission 430–480) signals were then detected using a Zeiss 710 Confocal Microscope (Carl Zeiss AG, Jena, Germany). For the LCI assay, tobacco leaves co-expressing the cLUC-ICE1 and LFR-nLUC constructs were incubated at 22°C for about 48 h. Then the luciferase substrate (1 mM D-luciferin) was sprayed onto the surface of the leaves and images were taken using a low-light cooled imaging apparatus (Fusion FX7; Vilber, Marne-la-Valle, France). The LUC activity was analyzed at 48 hpi using the NEWTONZ.0 Bio.Plant Imaging System (Vilber Bio lmaging, France).

### ChIP-qPCR assays

ChIP was performed as described previously (Yamaguchi et al., 2014). Briefly, 600 mg of 8-day-old seedlings were cross-linked using 1% formaldehyde; the reaction was stopped by the addition of 0.125 M glycine. The cross-linked chromatin was then sheared to an average size of ~500 bp, and 20 μL of the supernatant was used as the input control. Anti-FLAG antibodies (F3165; Sigma-Aldrich, St. Louis, MO, USA) were added to the remaining chromatin for immunoprecipitation followed by qPCR. Data are presented as the fold change relative to the wild type. The primers used are shown in **Supplementary Table S1**.

### Confocal microscopy and fluorescent signal quantification

Primary roots from 8-day-old *pLFR : LFR*-*enhancedYFP (eYFP)*/*lfr-*2 transgenic seedlings with or without cold treatment were stained with 10 μg/ml of propidium iodide (PI) for 5 min and then imaged using an Olympus FV 3000 Confocal Microscope (Olympus Corp., Tokyo, Japan) with the following settings: equal laser power, gain, and pinhole. Line scan analyses of the YFP and PI signals in the root tip were measured using Image J. For the relative eYFP fluorescence signals quantification, all the eYFP fluorescence signals in each picture were quantified by image J. Twenty roots were measured for each genotype. The signals for cold-treated roots are analyzed relative to the average fluorescence intensity of at time 0 h. Data were analyzed using Prism 8.0.

## Results

### Disruption of the SWI/SNF component LFR leads to hypersensitivity to cold stress

**Figure 1:**
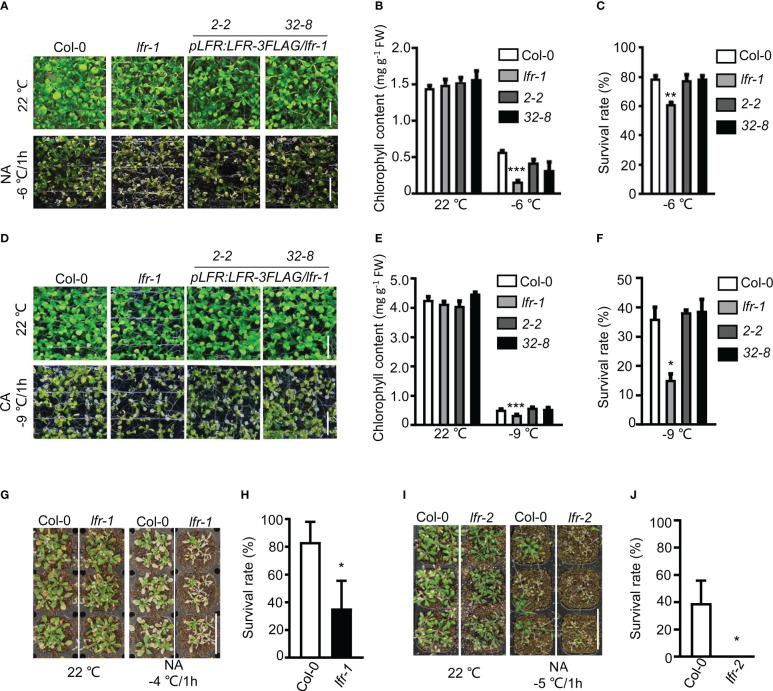
Phenotype analysis of *lfr* mutant plants and transgenic rescue lines exposed to freezing stress. **(A, D)** The *lfr-1* mutant is sensitive to freezing stress. Representative pictures of 10-day-old seedlings of Col-0, *lfr-1*, and two complementary lines of *pLFR : LFR*-3*FLAG*/*lfr-1* (2-2 and 32-8) were subjected to freezing stress under NA **(A)** or CA **(D)** conditions. After freezing stress, seedlings were incubated at 4°C overnight in the dark and then were allowed to recover for 2 days at 22°C. Scale bar=1 cm. **(B, E)** The chlorophyll contents of Col-0, *lfr-1*, and two complemented transgenic lines (2-2 and 32-8) treated with or without freezing stress under NA **(B)** and CA **(E)** conditions are shown. **(C, F)** The survival rates of Col-0, *lfr-1*, and two complemented transgenic lines (2-2 and 32-8) after being subjected to freezing stress under NA **(C)** or CA **(F)** conditions are shown. **(G)** and **(I)** Representative examples of the reduced freezing tolerance observed in soil-grown *lfr-1* **(G)** and *lfr-2* **(I)** plants. Scale bar=2 cm. **(H, J)** The survival rates of soil-grown Col-0 and *lfr-1* **(H)** and Col-0 and *lfr-2* **(J)** plants that were subjected to freezing stress (-4 or -5°C) and then allowed to recover are shown. Error bars indicate the SD of three biological replicates. Statistically significant differences are indicated by asterisks (*P<0.05, **P < 0.01, and ***P < 0.001; Student’s *t*-test).

To determine the function of the SWI/SNF component LFR in freezing tolerance, we evaluate the freezing tolerance phenotype of 10-day-old wild-type (WT, Col-0) and *lfr-1* seedlings grown on 1/2 MS medium under non-acclimated (NA) or cold-acclimated (CA) conditions. The NA seedlings were subjected to freezing stress at -6°C for 1 h. Under this condition, the *lfr-1* mutant showed reduced freezing tolerance with a significantly lower survival rate and chlorophyll content compared to WT after freezing treatment (**Figures 1A–C**). This defect was successfully rescued in *pLFR : LFR*-3*FLAG*/*lfr-1* transgenic lines, which showed no obvious difference compared to the WT control (**Figures 1A–C**). The CA seedlings were acclimated at 4°C for 4 days and then subjected to freezing stress at -9°C for 1 h. The *lfr-1* mutant also displayed freezing-sensitive defects under CA conditions (**Figures 1D–F**), and the defects were complemented in *pLFR : LFR*-3*FLAG*/*lfr-1* transgenic lines (**Figures 1D–F**). Thus, *LFR* is essential for freezing tolerance at the seedling stage under both CA and NA conditions.

To confirm the function of *LFR* in freezing tolerance, we subjected 3-week-old *lfr-1* and *lfr-2* (a strong sterile allele of *LFR*, segregated from *lfr-2+/-*) mutant plants to freezing stress in soil. The mutants were subjected to freezing stress at -4 or -5°C for 1 h and then returned to a growth chamber set at 22°C for 7 days. In terms of survival, *lfr-1* and *lfr-2* both displayed a freezing-sensitive phenotype (**Figures 1G–J**).

Taken together, these results indicate that *LFR* is a positive regulator of freezing tolerance in Arabidopsis.

### LFR is essential for cold-induced *CBFs* and *COR* expression in plants under cold stress

**Figure 2:**
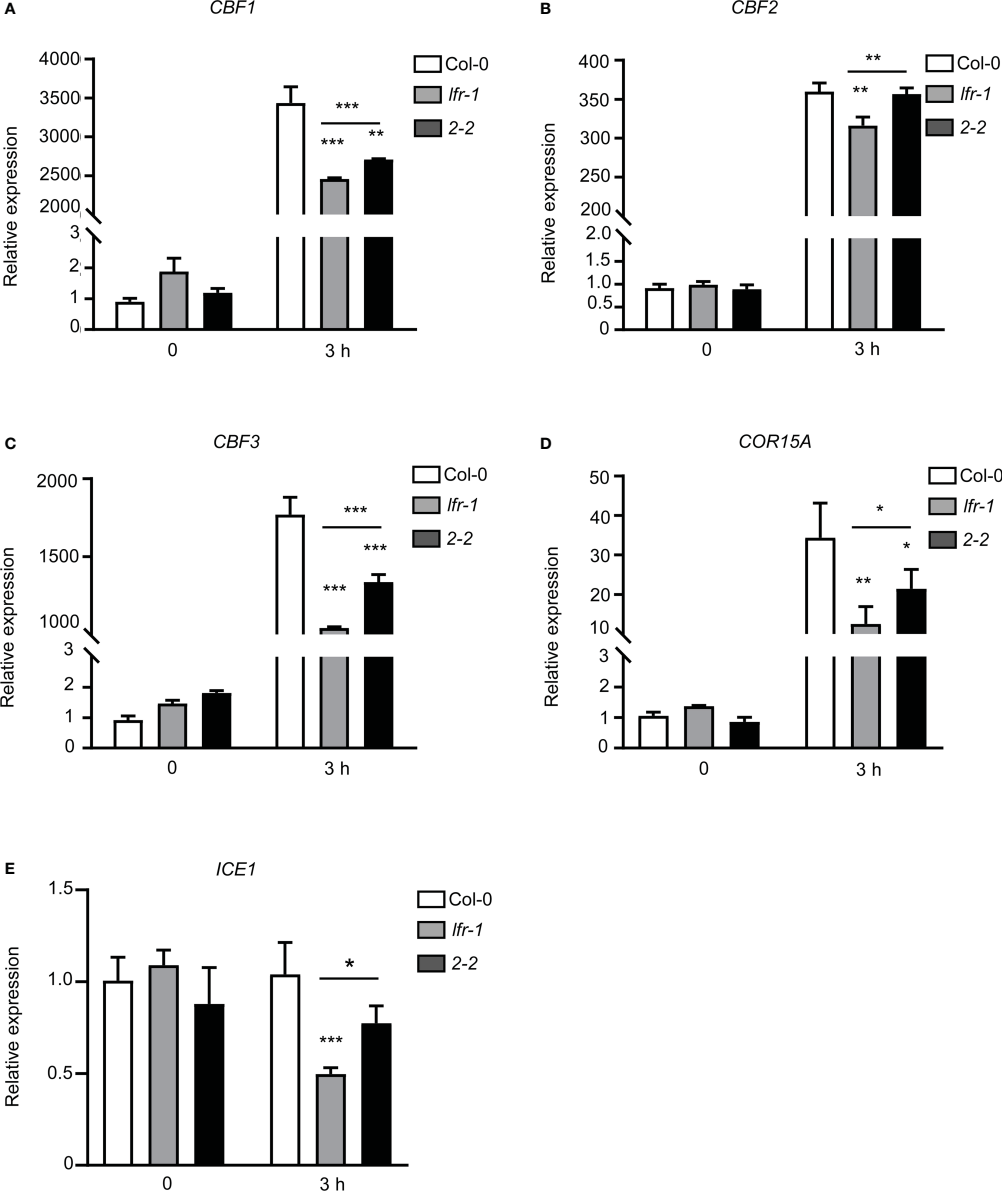
RT-qPCR analysis of *CBFs* and *COR* genes expression. **(A–E)** RT-qPCR data showing the transcript levels of *CBF1* **(A)**, *CBF2* **(B)**, *CBF3* **(C)**, *COR15A* **(D)**, and *ICE1* **(E)** in Col-0, *lfr-1*, and complemented transgenic line (2-2). Eight-day-old seedlings were treated at 4°C for the indicated time periods. Error bars indicate the SD of three biological replicates. Statistically significant differences are indicated by asterisks (*P < 0.05, **P < 0.01, and ***P < 0.001; Student’s *t*-test). *eIF4A1* was used as an internal control.

*ICE1*, *CBFs*, and downstream *COR* genes are important players in the response of plants to cold stress; thus, we next assessed the transcript levels of *ICE1*, *CBF1*, *CBF2*, *CBF3*, and *COR15A* in plants following low-temperature (4°C) treatment for 3 hours (3 h). Real time RT-qPCR showed that the expression levels of *CBF1*, *CBF2*, *CBF3*, and *COR15A* were significantly increased by cold treatment (**Figures 2A–D**). However, the expression levels of *CBF1*, *CBF2*, and *CBF3* were significantly reduced in *lfr-1* compared to Col-0 after cold treatment (**Figures 2A–C**). Similarly, the transcript level of *COR15A* was lower in *lfr-1* than in Col-0 after low-temperature treatment (**Figure 2D**). We also tested the expression levels of these four genes in *pLFR : LFR*-3*FLAG*/*lfr-1* complemented transgenic line (2-2), and we found that their expression levels were fully or partly rescued in the *lfr-1* mutant (**Figures 2A–D**). We also examined the expression level of *ICE1*, and found that it was not regulated by cold stress as reported previously (**Figure 2E**; Chinnusamy et al., 2003; Shi et al., 2018) *ICE1* was downregulated in *lfr-1* following cold treatment, and the downregulation of *ICE1* was partially rescued in complemented transgenic lines (**Figure 2E**). These results suggest that LFR is involved in cold acclimation *via* the direct or indirect transcriptional regulation of *ICE1*, *CBFs*, and *COR15A*.

### LFR interacts directly with ICE1 in yeast and plant

**Figure 3:**
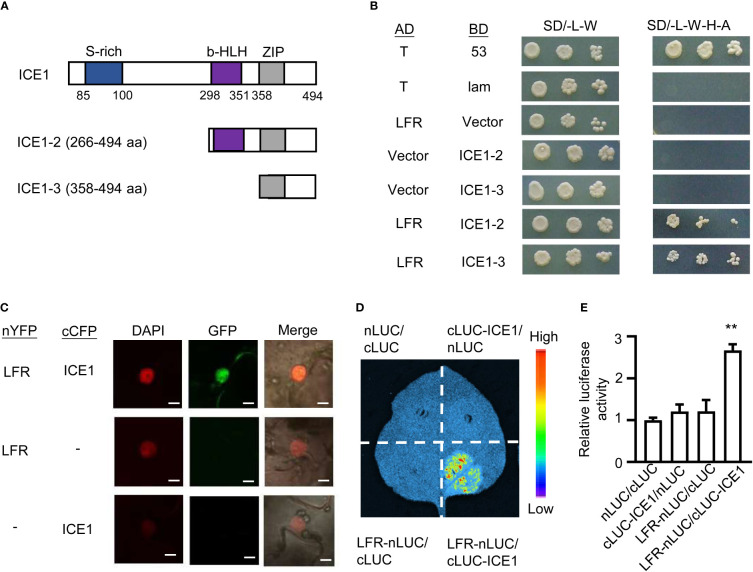
The interaction of LFR with ICE1. **(A)** Diagrams of the truncated ICE1 proteins used in the Y2H assays shown in **(B)**. ICE1-2 and ICE1-3 include the bHLH and ZIP (amino acids 298–494) and ZIP domains (amino acids 358–494), respectively. **(B)** LFR interacted with ICE1 in the Y2H assay. The assays were carried out using AH109 yeast cells co-transformed with the indicated plasmid pairs (serially diluted 10^-1^, 10^-2^, and 10^-3^) on synthetic defined medium lacking Ade, His, Leu, and Trp (SD/-L-W-H-A). AD-T/BD-53 and AD-T/BD-Lam were used as positive and negative controls, respectively. **(C)** nYFP-LFR interacted with cCFP-ICE1 in a BiFC assay carried out in transiently transformed tobacco leaf epidermal cells. Leaves co-transformed with nYFP-LFR/cCFP or nYFP/cCFP-ICE1 served as a negative control. DAPI and GFP fluorescence indicate the nucleus and protein interaction, respectively. Merge corresponds to an overlay of the DAPI and GFP signals. Bar =5 μm. **(D)** LCI assays were used to test for the interaction of LFR with ICE1 in transiently transformed tobacco leaf epidermal cells. Light emission was observed from tobacco leaves co-expressing LFR-nLUC/cLUC-ICE1, but not from leaves co-expressing the negative controls (LFR-nLUC/cLUC or cLUC-ICE1/nLUC or cLUC/nLUC). **(E)** The Relative luciferase activity of LCI assay in **(D)**. Error bars indicate the SD of four biological replicates. Statistically significant differences from the nLUC/cLUC control are indicated by asterisks (**P < 0.01, Student’s *t*-test).

To uncover the molecular mechanism underlying the role of LFR in cold acclimation, we examined Yeast two-hybrid (Y2H) data from a previous study (Lin et al., 2021). Interestingly, we noticed that ICE1 was isolated as an interacting partner of LFR. We confirmed this interaction by Y2H, Bimolecular Fluorescence Complementation (BiFC), and firefly luciferase (LUC) complementation imaging (LCI) assays. Because full-length LFR and ICE1 fused to the GAL4 BD showed obvious autoactivation in yeast (Ohta et al., 2018; Lin et al., 2021), truncated ICE1-2 (including the bHLH and ZIP domains) and ICE1-3 (including the ZIP domain) were fused to the BD to produce BD-ICE1-2 and BD-ICE1-3 (**Figure 3A**). We then co-transformed yeast strain AH109 with the AD-LFR and BD-ICE1-2 or BD-ICE1-3 plasmids. Yeast colonies co-transformed with AD-LFR/BD-ICE1-2 or AD-LFR/BD-ICE1-3 grew well on selective medium (SD/-L-W-H-A) (**Figure 3B**). However, negative control cells did not grow on the selective medium under the same conditions (**Figure 3B**). Thus, ICE1 probably interacts with LFR through its C-terminal ZIP domain.

We next conducted a BiFC assay using transiently transformed tobacco (*Nicotiana benthamiana*) leaves. GFP signals were detected in epidermal cell nuclei co-expressing nYFP-LFR and cCFP-ICE1; however, no GFP signal was detected in negative control cells co-expressing nYFP-LFR/cCFP or nYFP/cCFP-ICE1 (**Figure 3C**). These results indicate that LFR interacts with ICE1 in plant.

We next verified the LFR–ICE1 interaction using an LCI assay. Tobacco leaf areas co-transformed with LFR-nLUC and cLUC-ICE1 emitted light in the presence of luciferin. However, tobacco leaf areas co-transformed with the negative controls (LFR-nLUC/cLUC or cLUC-ICE1/nLUC or cLUC/nLUC) showed no light emission in the same condition (**Figures 3D, E**). These results confirm that LFR interacts with ICE1 in plant.

### LFR is associated with *CBF3* chromatin

**Figure 4:**
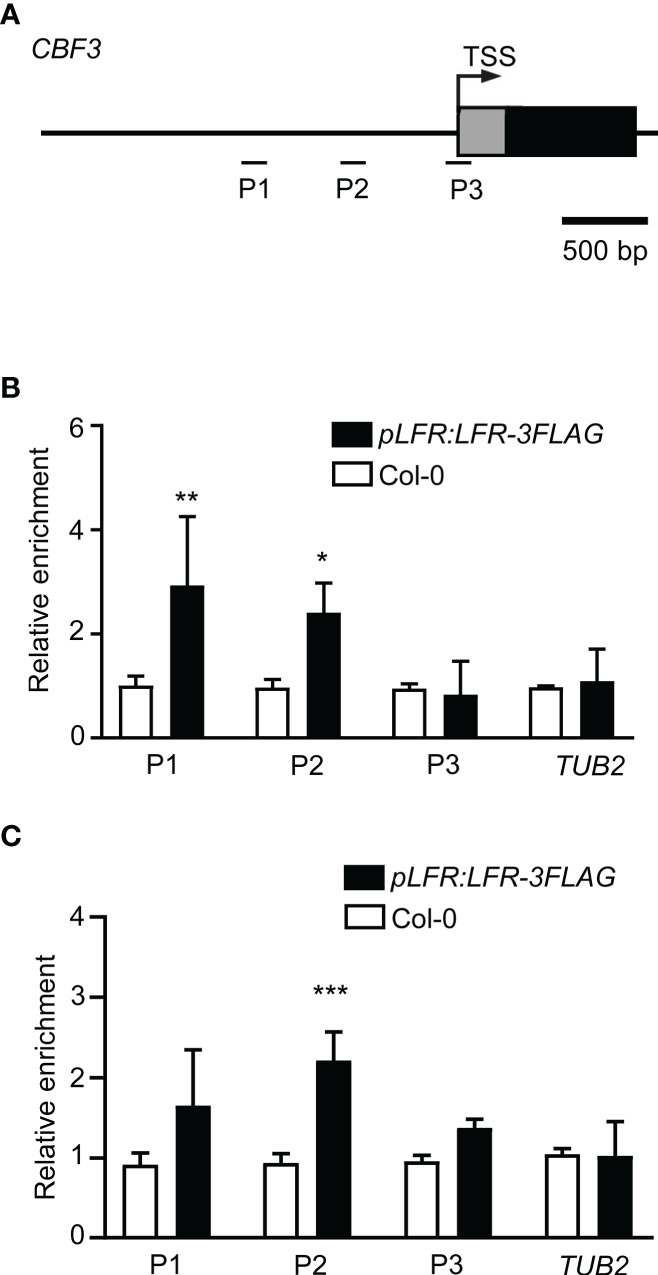
LFR binds to *CBF3* chromatin. **(A)** The gene structure of *CBF3*. Black boxes indicate exons; lines represent the promoter or intergenic region. Fragments amplified in the ChIP-qPCR assay are marked by short lines below the gene model. TSS, transcription start site. **(B, C)** ChIP-qPCR assay of LFR binding to *CBF3* without **(B)** and with low-temperature treatment **(C)**. Error bars indicate the SD of three biological replicates. *P < 0.05, **P < 0.01, ***P<0.001, Student’s *t*-test.

Given that *CBF3* is a direct target of ICE1 (Ding et al., 2015; Kim et al., 2015; Tang et al., 2020) and LFR interacts directly with ICE1, we speculated that LFR may also bind to the *CBF3* gene. ChIP-qPCR assays of *pLFR : LFR*-3*FLAG*/*lfr-2* complemented transgenic lines were conducted using anti-FLAG antibodies with Col-0 as a negative control. According to a previous report on the ICE1-binding site of *CBF3* (Ding et al., 2015), primers for the amplification of three different fragments (P1–P3) of *CBF3* chromatin were designed (**Figure 4A**). Under normal growth condition, the ChIP-qPCR result showed that LFR binds to *CBF3* at the P1 and P2 loci (**Figure 4B**). After low-temperature treatment, the ChIP-qPCR result showed that LFR binds to *CBF3* chromatin at the P2 locus (**Figure 4C**). The P1 and P2 loci of *CBF3* are also binding sites of ICE1 (Ding et al., 2015; Kim et al., 2015; Tang et al., 2020). Thus, these results demonstrated that *CBF3* is a direct target of LFR under normal and cold stress conditions.

### LFR is associated with *ICE1* chromatin

**Figure 5:**
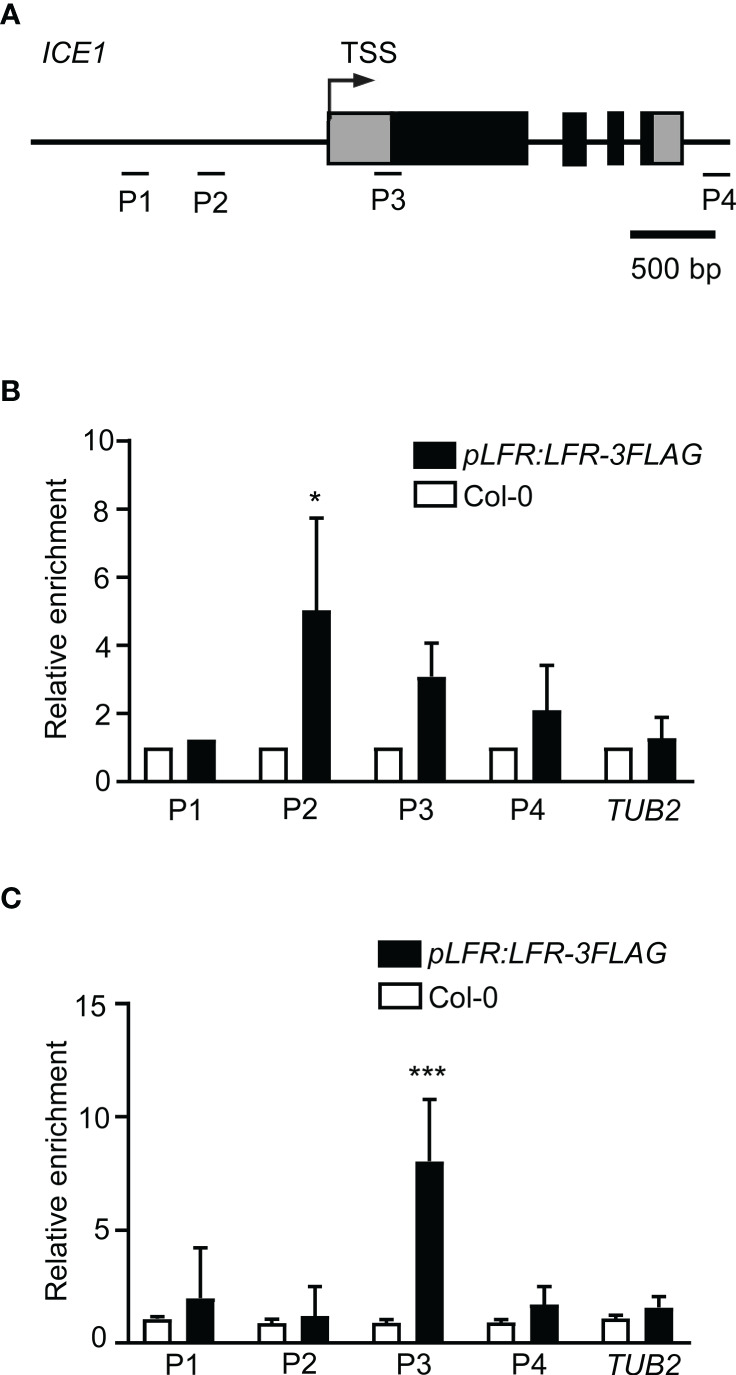
LFR binds to *ICE1* chromatin. **(A)** The gene structure of *ICE1*. Black boxes indicate exons; lines represent the promoter or introns. Fragments amplified in the ChIP-qPCR assay are marked by short lines below the gene model. TSS, transcription start site. **(B, C)** ChIP-qPCR assay of LFR binding to *ICE1* without **(B)** and with 4°C treatments **(C)**. Error bars indicate the SD of two (4°C) or three (22°C) biological replicates. *P < 0.05, ***P < 0.001, Student’s *t*-test.

Because we detected reduced expression of *ICE1* in *lfr-1* plants, we tested whether *ICE1* is also a direct target of LFR. A ChIP-qPCR assay was performed using Col-0 and the *pLFR : LFR*-3*FLAG*/*lfr-2* complemented transgenic line with anti-FLAG antibodies. Different primers for the amplification of different fragments of the *ICE1* locus were designed (**Figure 5A**). The ChIP-qPCR results showed that LFR was able to bind directly to the P2 locus of *ICE1* at 22°C (**Figure 5B**). After cold stress treatment, LFR was associated with the P3 locus of *ICE1* (**Figure 5C**). Thus, these results demonstrated that *ICE1* is also a direct target of LFR with or without low-temperature treatment.

### LFR is negatively regulated by cold stress

**Figure 6:**
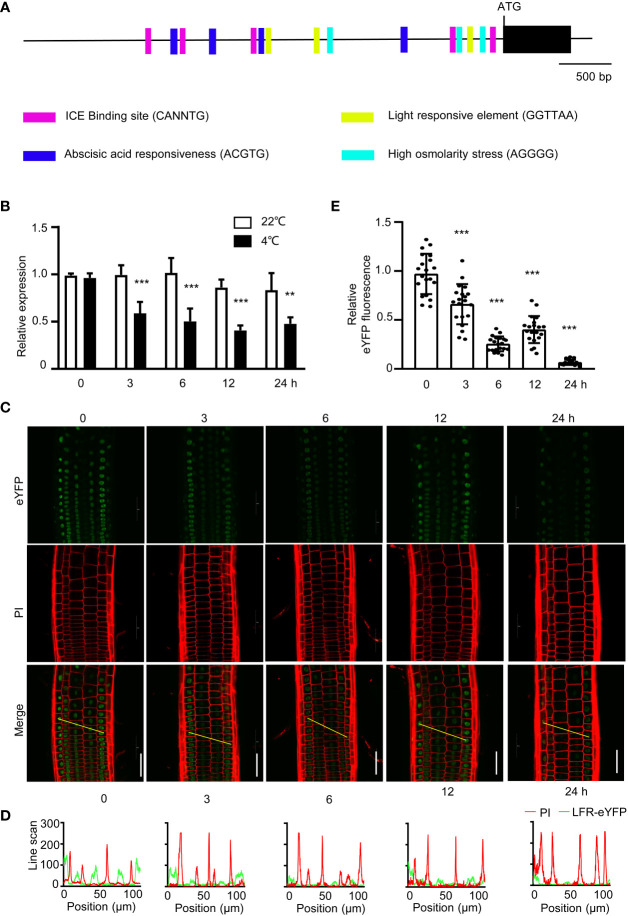
The expression of *LFR* are regulated by cold stress. **(A)** *Cis*-element analysis of the *LFR* promoter. Different *cis*-elements are indicated by different symbols. CANNTG is the low temperature-responsive element found in the ICE1 binding site. **(B)** RT- qPCR data showing the transcript level of *LFR* with (4°C) or without (22°C) cold treatment. RNA was isolated from 8-day-old Col-0 seedlings with or without cold treatment for 0, 3, 6, 12, or 24 h. *eLF4A1* served as an internal control. Error bars indicate the SD of three biological replicates. Statistically significant differences are indicated by asterisks. **P<0.01 and ***P<0.001, Student’s *t*-test. **(C)** Representative YFP signals observed in the roots of 8-day-old seedlings of *pLFR : LFR*-*eYFP*/*lfr-2* complemented transgenic lines as detected by confocal microscopy with (4°C, for 3, 6, 12, or 24 h) or without (0 h) cold treatment are shown. PI staining indicates the cell membrane. Scale bar= 50 μm. **(D)** Line scan analyses of the YFP (green line) and PI (red line) signals corresponding to the yellow lines indicated in **(C)** (Merge). YFP signals were significantly reduced after cold treatment. **(E)** Quantification of the YFP fluorescence in **(C)**. Representative data from one of three biological replicates with similar results are shown. Data are shown as the mean ± SD (n=20). ***P < 0.001; Student’s *t*-test.

To elucidate whether *LFR* is regulated by cold tress at the transcriptional level, we analyzed *cis*-regulatory elements in the LFR promoter using PLANTCARE. Five low temperature-responsive element (CANNTG) bound by ICE1 was found in the *LFR* promoter, indicating that *LFR* may be regulated by ICE1 or a cold-responsive gene (**Figure 6A**). And we indeed detected significant downregulation of *LFR* in *ice1-2* mutant plant (**Supplementary Figure S1A**). And we also checked the transcript level of *LFR* in plants treated with or without cold exposure at different time points. RT-qPCR showed that *LFR* was downregulated by cold treatment (**Figure 6B**). We also studied the protein level of LFR using *pLFR : LFR*-*eYFP*/*lfr-2* complemented lines. Furthermore, when the *pLFR : LFR*-*eYFP*/*lfr-2* lines were held at 4°C, the eYFP signals were reduced, especially after 6 and 24 h of cold stress (**Figures 6C–E**). Taken together, these results indicate that *LFR* may be repressed by cold stress.

## Discussion

### LFR is involved in the transcriptional regulation of the *ICE1–CBF–COR* cascade

Plants have developed sophisticated mechanisms to survive fluctuating ambient temperatures (Shi et al., 2018; Chen et al., 2021). At chilling or freezing temperatures, plants utilize a cascade of TFs to adapt to low-temperature stress (Shi et al., 2018). Among these TFs, ICE1, a constitutively expressed MYC-type TF, activates the expression of *CBFs*, which subsequently induce the transcription of downstream cold-responsive genes such as *CORs*, which promote cold tolerance (i.e., the *ICE1–CBF–COR* cascade) (Shi et al., 2018). However, the interacting factors that are needed for ICE1 to activate *CBFs* are largely unknown. In this study, we demonstrated that ICE1 is an LFR-interacting partner using Y2H, BiFC, and LCI assays (**Figure 3**). Disruption of LFR resulted in reduced cold tolerance under both NA and CA conditions, similar to *ice1-2* (**Figure 1**). In addition, the transcript levels of *ICE1–CBF–COR* cascade TF genes (e.g., *ICE1*, *CBF1*, *CBF2*, *CBF3*, and *COR15A*) were all downregulated after cold treatment in *lfr* plants (**Figure 2**). Like ICE1, LFR was able to directly bind to the *CBF3* locus (**Figure 4**). LFR was also found to associate directly with *ICE1* chromatin (**Figure 5**). Together, our results demonstrate that LFR directly interacts with ICE1 and positively regulates the transcription of genes in the *ICE1–CBF–COR* signaling pathway.

Interestingly, we also detected significant downregulation of *LFR* in *ice1-2* mutant plants, and we found ICE1-binding sites (i.e., CANNTG) in the proximal region of the *LFR* promoter (**Figure 6** and **Supplementary Figure S1A**). Furthermore, using published ChIP-seq data for GFP-ICE1 (Tang et al., 2020), we found that ICE1 was enriched at the proximal promoter region and gene body of *LFR* and *ICE1* chromatin (**Supplementary Figure S1**; Tang et al., 2020). Together with our data showing that ICE1 is not only an interacting factor but also a target of LFR, which tends to bind target(s) *via* interaction with transcription factor (Lin et al., 2018), these results may indicate that LFR and ICE1 interact physically and form transcriptional feedback loops. It will be interesting to test this possibility in the future.

Although LFR may act as a positive regulator of cold signaling, we found that *LFR* was repressed after prolonged cold treatment (**Figures 6**). This is similar to ICE1, which is degraded during long-term cold treatment (Dong et al., 2006; Ding et al., 2015; Zhao et al., 2017). The cold-induced downregulation of LFR and ICE1 may help balance plant cold responses and growth by preventing detrimental effects on plant growth resulting from the over-stimulation of critical freezing tolerance factors (e.g., CBFs and CORs) (Zhao et al., 2017).

In addition, we found that the *lfr-1 ice1-2* double mutant showed obvious defects in seed germination and greening (**Supplementary Figure S2**), which may indicate that *LFR* and *ICE1* may also participate in these developmental processes.

### The involvement of SWI/SNF CRC in low-temperature stress

Epigenetic factors play critical roles in long-term adaptation to the ambient environment (Miryeganeh, 2021; Chung et al., 2022). However, the exact functions of different CRC components in cold adaptation are unknown. Recently, Gratkowska-Zmuda et al. (2020) provided solid evidence showing that SWI3C of the SWI/SNF complex can directly bind, change the nucleosome occupancy and influence the transcription of *ICE1*, *MYB15*, and *CBF1* in a temperature-dependent manner. *SWI3C* was required to activate *ICE1* expression at 22°C but not at 14°C, and it repressed the transcription of all three *CBF* genes. Consistent with the upregulation of *CBFs*, the *swi3c* mutant displayed increased freezing tolerance compared to wild type (Gratkowska-Zmuda et al., 2020). However, in this study, we found that *ICE1*–*CBF*–*COR* cascade genes were downregulated in the *lfr* mutant, which displayed hypersensitivity to freezing. Our previous study showed that LFR interacts with SWI3A/SWI3B, but not SWI3C (Lin et al., 2021). Consistently, LFR together with SWI3A/SWI3B, not SWI3C, were identified in the immunoprecipitation mass spectrometry (IP-MS) data of a recent study of the MINU-associated SWI/SNF (MAS) complex (Diego-Martin et al., 2022; Hernández-García et al., 2022). In addition, a comprehensive characterization of the SWI/SNF complexes by proteomic approaches revealed that SWI3C and LFR are involved in different complexes with specific chromatin accessibility and binding ability for active histone modification (Guo et al., 2022). Thus, the opposite phenotype and differential expression pattern of *CBF* genes may result from that SWI3C and LFR participate in distinct SWI/SNF complexes with different transcriptional properties. So, it will be interesting to decipher the detailed molecular and genetic interactions between LFR, SWI3C, and the core ATPases of the SWI/SNF complex (BRM, SYD, MINU1, and MINU2), which is of great importance to elucidate the molecular regulatory mechanisms of SWI/SNF complexes in controlling plant response to cold treatment.

Arabidopsis *LFR* encodes an ARM-repeat domain-containing protein (Wang et al., 2009). Phylogenetic analysis of the ARM domain revealed that plant LFR has a common origin with the signature subunits ARID1/2 of the animal SWI/SNF complex (Hernández-García et al., 2022). Recently, the structural approaches demonstrated that the ARM domain in ARID1 serves as a rigid core in the “Base/Core module” organization and stabilization by directly interacting with the core ATPase and all other Base subunits (He et al., 2020; Mashtalir et al., 2020). Besides, ARID1 is also essential for the efficient nucleosome sliding activity of the SWI/SNF complex (He et al., 2020). However, the molecular weight of Arabidopsis LFR is much lower than its mammalian orthologs, because of the loss of the DNA-interacting AT-rich interaction domain (ARID) (Wang et al., 2009; Hernández-García et al., 2022). Thus, it will be interesting to decipher the biological contribution of plant LFR, to the complex organization, architecture, stability, and nucleosome sliding activity of target genes in plant development and stress in the future.

**Figure 7:**
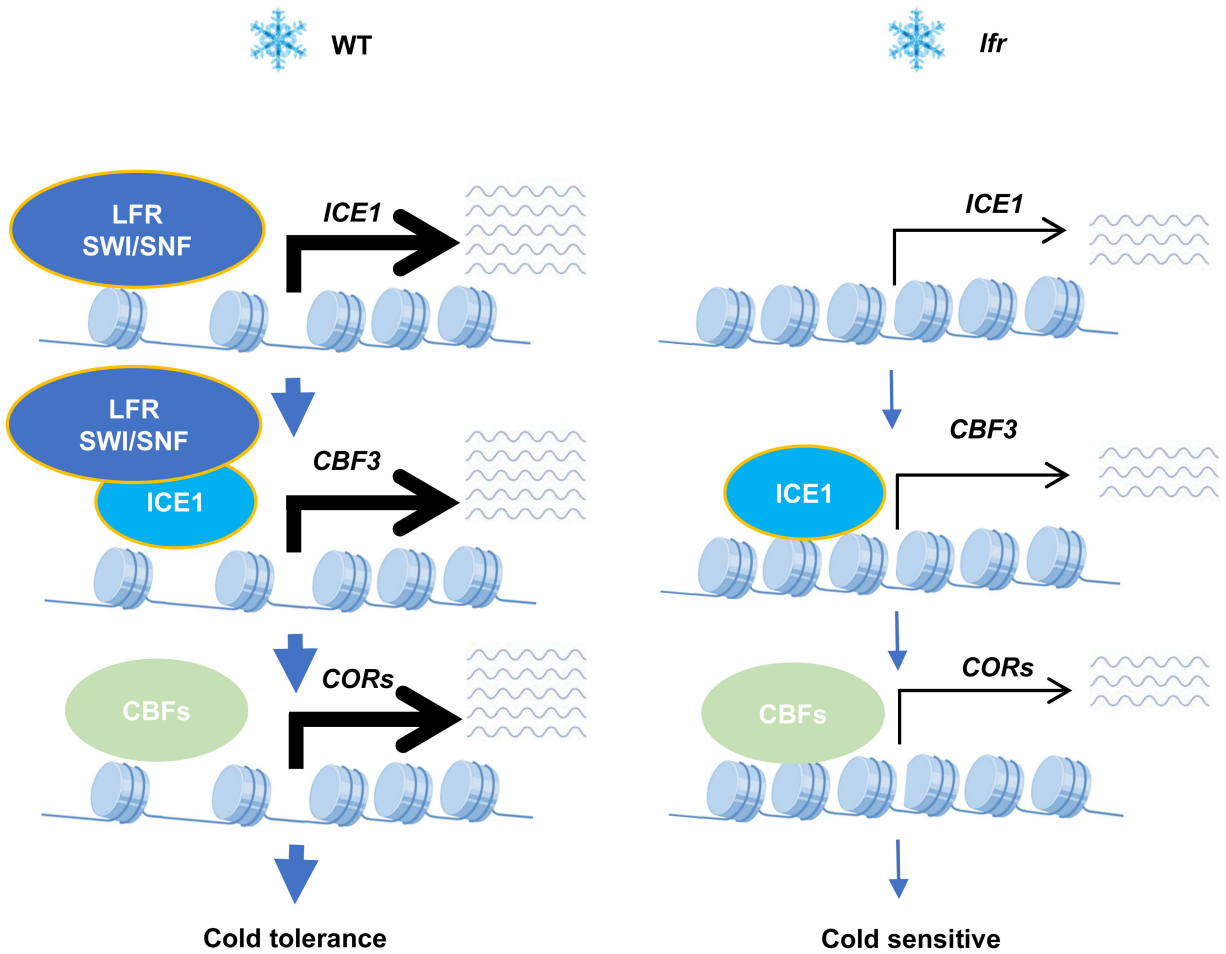
A working model of the function of the SWI/SNF subunit LFR in cold acclimation. Under low-temperature conditions, the SWI/SNF complex component LFR promotes plant survival by acting as a positive regulator of the *ICE1*–*CBF*–*COR* cascade through its physical interaction with ICE1. The depletion of *LFR* results in the downregulation of *ICE1*–*CBF*–*COR* cascade genes and cold-sensitive defects.

Taken together, our results demonstrate that the SWI/SNF complex component LFR interacts physically with ICE1 to act as a positive regulator of the *ICE1*–*CBF*–*COR* cascade (**Figure 7**). This study will aid our understanding of plant-specific epigenetic changes through chromatin remodeling in response to global climate change.

## Conclusion

Our findings indicate that the SWI/SNF ATP-dependent CRC component LFR interacts directly with ICE1, and that LFR plays a positive regulatory role in the transcription of *ICE1*–*CBF*–*COR* cascade genes. LFR can bind directly to the promoter regions of *ICE1* and *CBF3* to regulate their expression and thus participate in the response of plants to low-temperature stress. These results suggest that ICE1 may promote plant survival at freezing temperatures by interacting with the SWI/SNF CRC to activate target gene expression. Our findings provide important information for breeding climate-resilient crops.

## Accession numbers

Data were deposited in TAIR (Arabidopsis Information Resource) under accession numbers: LFR (AT3G22990), ICE1 (AT3G26744), CBF1 (AT4G25490), CBF2 (AT4G25470), CBF3 (AT4G25480), COR15A (AT2G42540).

## Data availability statement

The datasets presented in this study can be found in online repositories. The names of the repository/repositories and accession number(s) can be found in the article/**Supplementary Material**.

## Author contributions

SC and HZ designed the study. TM constructed the plasmids and performed the Y2H, BiFC, LCI, ChIP-qPCR, phenotype analysis, and RT-qPCR assays. SW and JT conducted the ChIP-qPCR, RT-qPCR, and protein level analyses. CS did the phenotype analyses. HG prepared the *pLFR : LFR*-*3FLAG* and *pLFR : LFR*-*eYFP* transgenic lines. SW, HZ, and SC wrote the manuscript with input from all the co-authors. All authors contributed to the article and approved the submitted version.
