# Supplementary material for: Arabidopsis LFR, a SWI/SNF complex component, interacts with ICE1 and activates ICE1 and CBF3 expression in cold acclimation
Source: Front Plant Sci. 2023 Mar 21;14:1097158. doi: 10.3389/fpls.2023.1097158 (PMC10070696; doi:10.3389/fpls.2023.1097158)
Supplement: Supplementary file 1 [file DataSheet_1.pdf]

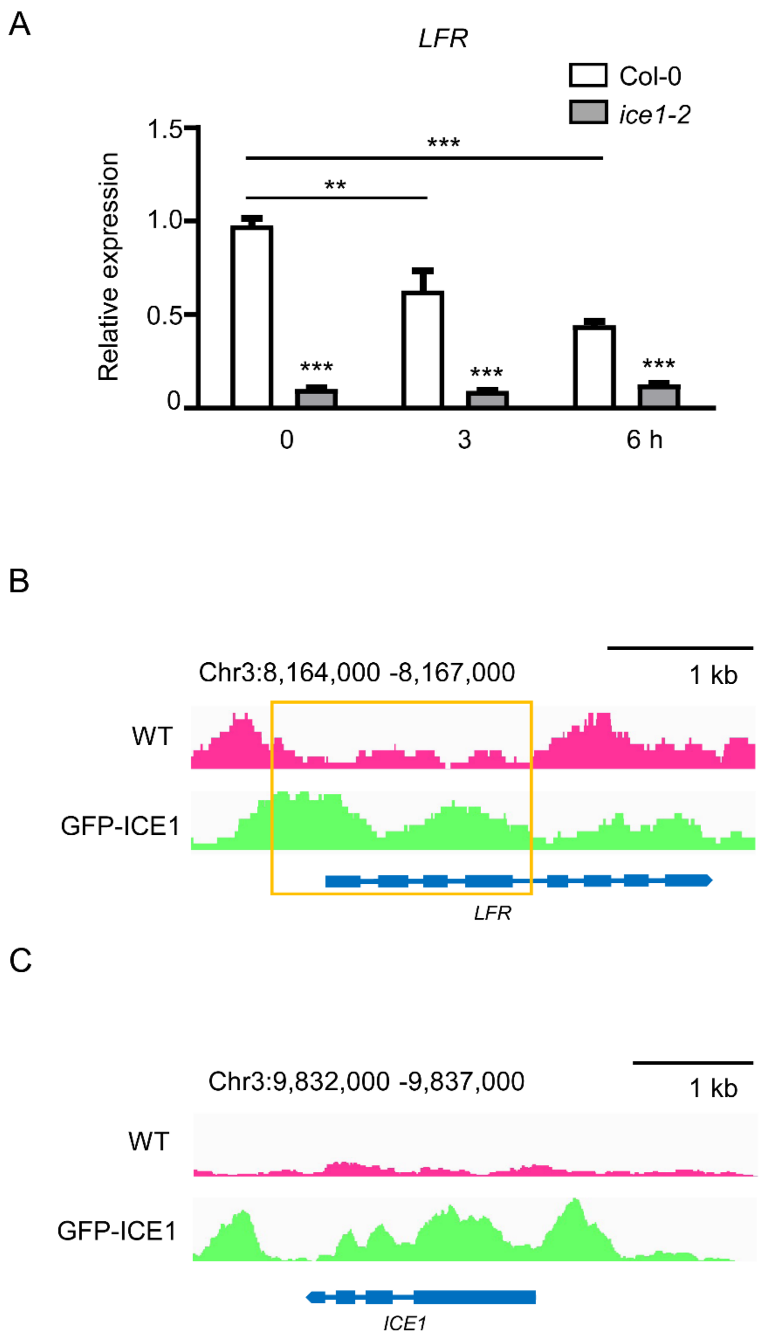


**Supplementary Figure 1** ICE1 may regulate the expression of *LFR*.

**(A)** RT-qPCR data showing the transcript levels of *LFR* in Col-0 and *ice1-2* plants. Eight-day-old seedlings were treated at 4°C for 0, 3, or 6 h. Error bars indicate the SD of two biological replicates. Statistically significant differences are indicated by asterisks (**$P<0.01$ and ***$P<0.001$; Student's *t*-test). *eIF4A1* was used as an internal control.

**(B) and (C)** The possible enrichment of GFP-ICE1 on *LFR* chromatin (A, indicated by the yellow box) and *ICE1* chromatin (B). Our analysis was conducted using ChIP-seq data for GFP-ICE1 from (Tang et al., 2020).

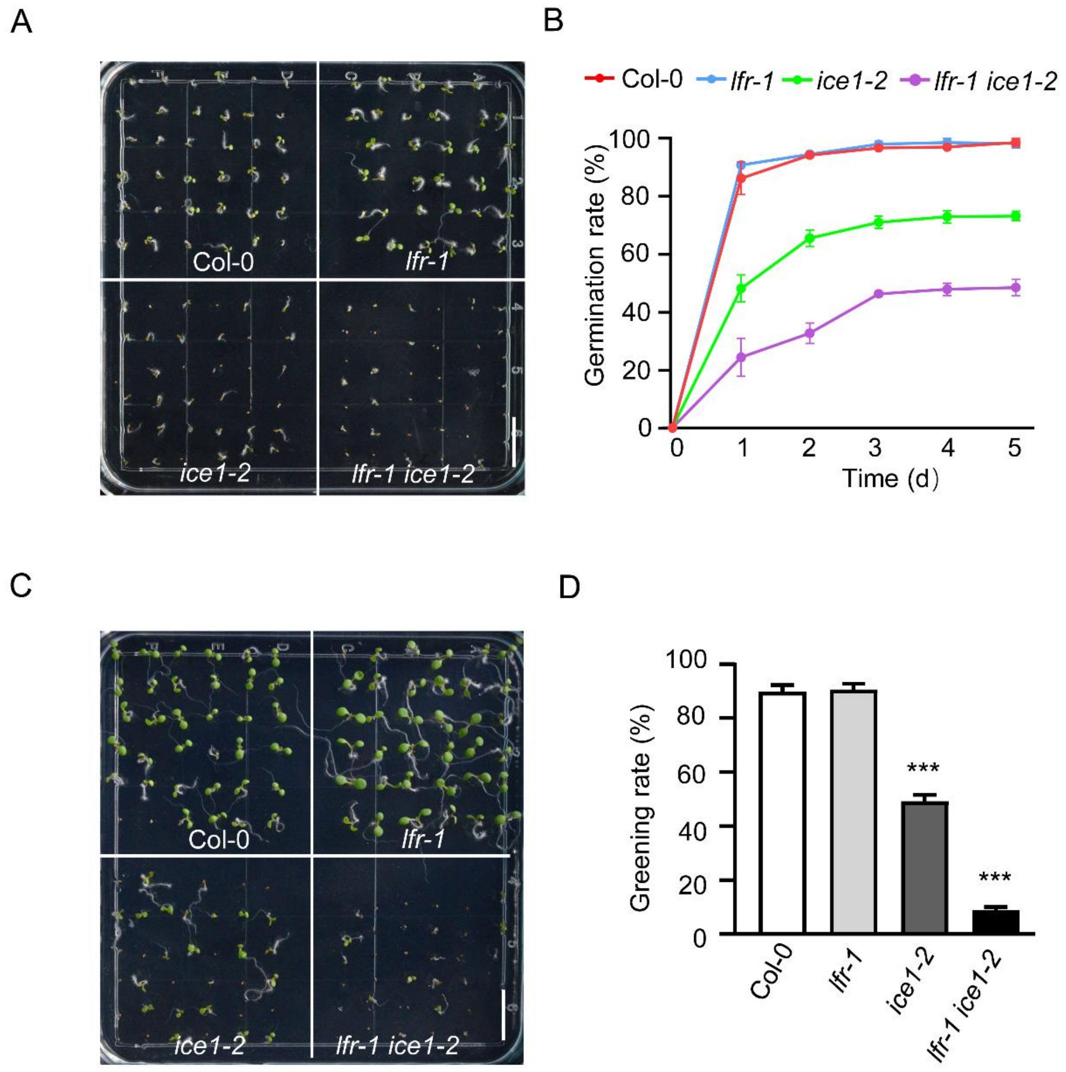


**Supplementary Figure 2** Seed germination and greening phenotypes of Col-0, *lfr-1*, *ice1-2* single and double mutants.

(A) The seed germination phenotype of the 3-day-old Col-0, *lfr-1*, *ice1-2*, and *lfr-1 ice1-2* plants.

(B) The seed germination rates were analyzed at the indicated time (days) for the Col-0, *lfr-1*, *ice1-2*, and *lfr-1 ice1-2* plants.

(C) The greening phenotype of the 5-day-old Col-0, *lfr-1*, *ice1-2*, and *lfr-1 ice1-2* plants.

(D) The greening rates of the 5-day-old Col-0, *lfr-1*, *ice1-2*, and *lfr-1 ice1-2* plants. Error bars indicate the SD of three biological replicates. Statistically significant differences are indicated by asterisks (***P<0.001; Student's *t*-test).

Supplementary Table 1 | The primers used in this study

| Primer | Sequence (5'→3') | Description |
|---|---|---|
| ICE1-P1 | FP:TGGCTGTTTGTACCAATCCACAT | ChIP-qPCR |
| | RP:AAACTCGGATTTACTCTAATCGCG | |
| ICE1-P2 | FP:GAAGACTAGAAAGCATATCGCAACA | |

| | | |
|---|---|---|
| | RP:CGACATCTTTTCGATGGTTCCTA | |
| ICE1-P3 | FP:CTTCTTCTTCTTGTTCTCCTTCTCAAGC | |
| | RP:AACCAAACCCCATCGAAATAGGAGC | |
| ICE1-P4 | FP:TCAAAGAAGACAGACAAAACATTTGCA | |
| | CAT | |
| TUB2 | RP:TGCTCTGCCTCTTCCTTCACCA | |
| | FP:ATCCGTGAAGAGTACCCAGAT | |
| CBF3-P1 | RP:AAGAACCATGCACTCATCAGC | |
| | FP:AAAGTCTTCTCTGGACACATGG | |
| CBF3-P2 | RP:AAAGGCGAAGACGGAGTTT | |
| | FP:GTTACATTTGATCATTCACCCA | |
| CBF3-P3 | RP:GGGAGAGTAGATATTTGTGCAAC | |
| | FP:CCATTTGTTAATGCATGATGGTAGA | |
| CBF1 | RP:GAAGCATGCAGTTGTAATTGTAGT | |
| | FP: GCATGTCTCAACTTCGCTGA | |
| CBF2 | RP:ATCGTCTCCTCCATGTCCAG | RT-qPCR |
| | FP:TGACGTGTCCTTATGGAGCTA | |
| CBF3 | RP:CTGCACTCAAAAACATTTGCA | |
| | FP:GATGACGACGTATCGTTATGGA | |
| ICE1 | RP:TACACTCGTTTCTCAGTTTTACAAAC | |
| | FP:GGGTTTGCCTTGGATGTTTT | |
| LFR | RP:ATCATACCAGCATACCCTGC | |
| | FP: CTGTTGGAGCACTCTACAATCTCG | |
| | RP: GGATGCGGAGTCTTTATCACTTTC | |
